# Community and provider perceptions of traditional and skilled birth attendants providing maternal health care for pastoralist communities in Kenya: a qualitative study

**DOI:** 10.1186/s12884-016-0828-9

**Published:** 2016-03-01

**Authors:** Abbey Byrne, Tanya Caulfield, Pamela Onyo, Josephat Nyagero, Alison Morgan, John Nduba, Michelle Kermode

**Affiliations:** Nossal Institute for Global Health, University of Melbourne, 161 Barry St, Carlton, VIC 3010 Australia; Amref Health Africa, PO Box 27691-00506, Nairobi, Kenya

**Keywords:** Maternal health, Newborn health, Skilled birth attendants, Traditional birth attendants, Health systems, Pastoralist communities, Kenya

## Abstract

**Background:**

Kenya has a high burden of maternal and newborn mortality. Consequently, the Government of Kenya introduced health system reforms to promote the availability of skilled birth attendants (SBAs) and proscribed deliveries by traditional birth attendants (TBAs). Despite these changes, only 10 % of women from pastoralist communities are delivered by an SBA in a health facility, and the majority are delivered by TBAs at home. The aim of this study is to better understand the practices and perceptions of TBAs and SBAs serving the remotely located, semi-nomadic, pastoralist communities of Laikipia and Samburu counties in Kenya, to inform the development of an SBA/TBA collaborative care model.

**Methods:**

This descriptive qualitative study was undertaken in 2013–14. We conducted four focus group discussions (FGDs) with TBAs, three with community health workers, ten with community women, and three with community men. In-depth interviews were conducted with seven SBAs and eight key informants. Topic areas covered were: practices and perceptions of SBAs and TBAs; rewards and challenges; managing obstetric complications; and options for SBA/TBA collaboration. All data were translated, transcribed and thematically analysed.

**Results:**

TBAs are valued and accessible members of their communities who adhere to traditional practices and provide practical and emotional support to women during pregnancy, delivery and post-partum. Some TBA practices are potentially harmful to women e.g., restricting food intake during pregnancy, and participants recognised that TBAs are unable to manage obstetric complications. SBAs are acknowledged as having valuable technical skills and resources that contribute to safe and clean deliveries, especially in the event of complications, but there is also a perception that SBAs mistreat women. Both TBAs and SBAs identified a range of challenges related to their work, and instances of mutual respect and informal collaborations between SBAs and TBAs were described.

**Conclusions:**

These findings clearly indicate that an SBA/TBA collaborative model of care consistent with Kenyan Government policy is a viable proposition. The transition from traditional birth to skilled birth attendance among the pastoralist communities of Laikipia and Samburu is going to be a gradual one, and an interim collaborative model is likely to increase the proportion of SBA assisted deliveries, improve obstetric outcomes, and facilitate the transition.

## Background

Traditional birth attendants (TBAs) are lay community members, mostly women, who assist with childbirth, and who are commonplace in many low-income settings. The story of TBAs in global maternal health programming has been characterised by both engagement and exclusion. In the 1970s and 1980s the efforts to reduce maternal mortality focused on training TBAs to provide safer delivery care. However, the impact of this strategy on maternal mortality was negligible as TBAs are not equipped to manage maternal complications, and risk screening for complicated deliveries is poorly predictive of outcomes [1]. Consequently, the focus in the 1990s was on scaling up access to skilled birth attendants (SBAs), who are trained health workers such as nurses, midwives and doctors, able to manage complicated deliveries and resuscitate newborn babies, and who mostly work in health facilities [2]. The shift to SBAs as the preferred provider of maternal health care was clearly reflected in the Millennium Development Goals, which nominated the proportion of deliveries attended by SBAs as the primary indicator of progress in maternal mortality reduction [3]. The practices of TBAs were subsequently ignored, and in some places even proscribed by government. For complex reasons, many women in low-income settings are still cared for by TBAs rather than SBAs at the time of pregnancy and delivery [4–6]. TBAs are valued by communities because they are accessible, inexpensive, and well-respected; abide by local traditions; and provide services that SBAs do not, such as in-home postpartum care [4–10]. In recent years there has been some reassessment of the TBA role, and the potential for SBAs to collaborate with them to improve maternal and newborn health outcomes [10, 11].

Kenya experiences a high burden of maternal and newborn mortality with a maternal mortality ratio of 488 deaths per 100,000 live births (95 %CI 333, 643), and a neonatal mortality rate of 30 deaths per 1000 live births [12, 13]. As these rates were not improving with the passage of time, the Government of Kenya instituted health system reforms aiming to have 90 % of deliveries assisted by SBAs by 2015, a doubling from the 2008 level of 43 % [12]. To achieve this goal, beginning in 2009, the Government deployed SBAs to level two health facilities (commonly referred to as dispensaries), which are mostly located in remote areas such as those populated by pastoralist communities. The Government also actively discourages TBA supported births. However, despite these initiatives, uptake of SBA services remains low, and inequitably distributed; for example, 89 % of women giving birth in Nairobi were attended by an SBA during 2008–09, while fewer than one in ten women birthing in remote pastoralist communities had an SBA present in 2012 [12, 14]. A 2012 survey among the remote semi-nomadic pastoralist communities of Laikipia and Samburu counties in the Rift Valley region revealed that 92 % of births took place at home, and 57 % were assisted by a TBA. The remaining home deliveries were attended by family members or the woman delivered alone [14].

Providing women in remote semi-nomadic pastoralist communities with maternal health care from SBAs is particularly challenging. Barriers include: more remote settings with fewer health facilities; mobile populations for whom static health services are less accessible; and strong traditions of using TBAs [10].

The Government proscription of TBA assisted deliveries means that health planners no longer engage with TBAs, so little is understood about their current roles and practices in remote communities. The objectives of this qualitative study were to investigate and describe the: 1) roles and practices of TBAs and SBAs serving pastoralist communities in Laikipia and Samburu counties, Kenya; 2) attitudes of TBAs and SBAs regarding their own practices and those of one another; 3) communities’ attitudes in relation to care provided by TBAs and SBAs.

## Methods

In order to better understand practices and perceptions of TBAs and SBAs providing maternal care in remote pastoralist communities in Kenya we undertook a descriptive mixed methods study. This paper reports on the qualitative phase of the study.

### Study setting

This work was undertaken by a partnership between the Nossal Institute for Global Health at the University of Melbourne, Amref Health Africa, the Mothers Union of the Anglican Church in Kenya (MUACK), and the relevant County Health Ministries. The study builds on the existing health and development projects of MUACK with the pastoralist communities of Laikipia and Samburu counties of Kenya. These pastoralist counties are sub-divided into group ranches that consist of several villages, and each of these consist of a small number of homesteads (*manyattas*). The study sites were five group ranches in Laikipia (Chumvi, Morupusi, Makurian, Naibor, Tiamamut), and three in Samburu (Kirimon, Kisima, Longewan). Participating communities are mostly semi-nomadic; men migrate with cattle during dry seasons while women and children remain at home for most of the year caring for goats and sheep. The predominant language is Maa, although Kiswahili, English and other tribal languages and dialects of Maa are spoken. The health system comprises: dispensaries located in group ranches, which serve as primary care facilities mostly staffed by solo nurses; health centres and sub-district hospitals where medical officers and basic emergency obstetric care can be provided; and the district hospital where comprehensive emergency obstetric care is available.

### Sampling

The qualitative data collection involved: focus group discussions (FGDs) with TBAs, community health workers (CHWs), and community women and men (Table 1). CHWs are local women and men who have received brief intensive training as part of the Ministry of Health’s ‘Community Strategy’; each group ranch has its own CHWs. A component of their role is to link pregnant women with health facilities. CHWs are ideally positioned to understand the perspectives of communities, TBAs and SBAs, hence their inclusion in the study. The number and range of FGDs was anticipated to be sufficient to achieve data saturation. We conducted individual semi-structured in-depth interviews (IDIs) with SBAs and key informants. IDIs were conducted with the seven SBAs (all dispensary level nurses) from the study sites because it was not logistically feasible to bring them all together in one place for an FGD; most would have to travel a long distance to participate in an FGD, which would involve leaving the dispensaries unattended. Purposive sampling was used to select the group ranches for the various FGD categories based on background knowledge of the sites. Specifically, the sampling approach ensured inclusion of people residing in places with and without a local health facility and SBA, and people who were close to and far away from health facilities. Sampling the FGD participants from within each study site was largely opportunistic.

**Table 1:** Summary of FGD participants and content

Category	Number FGDs	Total no. participants	Topic areas investigated during FGDs
Traditional birth attendants (TBAs)	4	46	Experience & training; perceptions of quality care; rewards & challenges; managing complications; community preferences for care; options for TBA/SBA collaboration.
Community health workers (CHWs)	3	45	Community preferences for care; reasons for this; decision making about care; accessibility of services; managing complications; perceptions of TBAs and SBAs.
Women who have delivered with an SBA in the past two years	3	27	Care seeking choices and decision making; practitioner preferences; reasons for this; experiences of care; characteristics of good quality care; options for SBA/TBA collaboration.
Women who have delivered with a TBA in the past two years	5	59	As above.
Women who have delivered unattended in the past two years	2	30	As above.
Husbands of women who have delivered in the past two years	2	25	Care seeking choices and decision making; practitioner preferences; reasons for this; characteristics of good quality care; options for SBA/TBA collaboration.

A total of seven IDIs were conducted with SBAs (five female and two male; all were based in dispensaries) and eight IDIs with key informants i.e., dispensary managers (2), staff from the County Departments of Health (2), and male (2) and female (2) Community Development Committee (CDC) members. The topic areas discussed during the SBA interviews were similar to those identified for the FGDs with TBAs (see Table 1); and the topic areas for the key informant interviews were roles and relationships of SBAs and TBAs, and options for improved collaboration.

**Data collection** was undertaken from October 2013 to March 2014. The project commenced with engagement of stakeholders and permission from community leaders to proceed with the study in each of the group ranches. Interview guides for the FGDs and IDIs were drafted in English by the researchers based on the study objectives and the literature, and reviewed with the local data collection team who had in-depth knowledge of the communities. Interview guides were translated into Maa or Kiswahili language by local research team members, and back translated to English, piloted and revised. Pictures representing pregnancy, birth, birth complications, and the different types of health facilities were created by a local artist and used as prompts to stimulate discussion during the FGDs with TBAs and community women.

Data were collected by local research team members (two females, one male; two Maa speakers, one Kiswahili speaker) trained in FGD facilitation and IDI techniques, and supervised by study investigators. FGDs and IDIs were conducted in Maa, Kiswahili and English languages dependent on the preference of the participants. Participants were reimbursed for transport and lunch expenses, typically to the value of 400 Kenyan Shillings (approx. USD 4), consistent with the standard payment dispensed by NGOs active in the sites. All FGDs and IDIs were digitally audio-recorded.

### Data analysis

All FGDs and IDIs were translated and transcribed verbatim by local team members and cross-checked for accuracy. The transcriptions were inductively and deductively thematically analysed by two of the researchers independently (AB & TC), and subsequently assessed for consistency and divergence [15]. Analysis was undertaken manually by one researcher, and using NVivo 10 software by the other. The final themes were those that emerged from the data i.e., what participants said in response to questions from the interview guide, questions asked in order to probe responses, and questions asked to obtain clarity on what was being said. Our approach was more emic than etic in orientation. Following data analysis, interim findings were shared with representatives of group ranches to ensure that they resonated with the varied experiences and perceptions of the communities. The topic areas and themes relevant to this paper are summarised in Table 2.

**Table 2:** Themes that emerged from FGDs, semi-structured interviews, and key informant interviews

Topic area	Themes
TBA practices	
- antenatal	Dietary and workload advice; abdominal massage; linking with SBAs.
- delivery	Receiving the baby; giving comfort to mother; mediating with the husband; administration of special foods and herbs; abdominal massage; observing traditions.
- post-partum	Keeping mother and baby warm; preparing special foods; cleaning the woman; disposal of placenta; assistance with domestic chores; encouraging breast feeding.
- complications	Administration of herbs; food supplementation; inducing vomiting; referral to health facility.
- challenges	Fear of becoming infected with HIV when women are ‘sick’; managing complications; difficulty obtaining transport for referrals
SBA practices	
- antenatal	Checking vital signs of woman and baby; administration of medications/immunisation; making a birth plan; referral for laboratory testing; dietary and workload advice; promotion of bed nets.
- delivery	Monitoring the mother and baby; encouraging the woman.
- post-partum	Monitoring baby and mother; care of episiotomy/tear; encouraging breastfeeding; dietary advice; family planning.
- complications	Transfer to higher level facility.
- challenges	Trying to get women to attend health facility for delivery; doing deliveries at home; difficulty getting an ambulance in case of complications; lack of necessary equipment at health facility; being a lone practitioner.
Perceptions of TBAs	
- strengths	Accessible and available; assistance with domestic chores; referral and accompanying to health facility; trusted and respected by community; valued traditional knowledge; courage.
- concerns	Inability to manage complications; poor hygiene; unsafe practices.
Perceptions of SBAs	
- strengths	Valuable technical knowledge & skills; provision of safety; access to equipment, medications & injections; ability to manage complications; able to refer easily.
- concerns	Negative attitudes and behaviours towards women; leaving women alone; cold facilities; unwanted clinical procedures; staff absenteeism; restricted visitors; lack of equipment.

### Ethics

Ethics approval was granted in June 2013 from the Ethics and Scientific Review Committee of Amref Health Africa Kenya, and the Human Research Ethics Committee of The University of Melbourne, Australia. Informed consent was obtained from all participants, using written or verbal methods dependent on the participant’s level of literacy.

## Results

### Practices of TBAs

Traditional birth attendants are predominantly older women who gain their skills through experience and working alongside other TBAs. In this way, knowledge is passed across generations. TBAs are seen as the repository of knowledge regarding traditional birth practices and associated herbal treatments. Additionally, they mediate between the pregnant or labouring woman and her husband, provide advice about care required, including diet, when to slaughter an animal, and if referral to a health centre is necessary.

*The woman tells the TBA what she wants, she cannot tell the husband. I [the TBA] also ask her if there is anything she needs, then I tell the husband. If she needs meat, then we* [TBAs] *tell the husband and a goat is slaughtered.* [FGD with TBAs, Laikipia]

#### Antenatal care by TBAs

TBAs advise women to reduce their workload to avoid miscarriage during the early stages of pregnancy, and in some group ranches this advice extends throughout pregnancy. In many but not all group ranches, TBAs and other women assist pregnant women in their first trimester with their domestic chores such as collecting firewood, cooking, and washing clothes. They also recommend that women eat foods of their choosing at this stage of pregnancy, although advice concerning the quantity of food intake varied; some TBAs said that women should avoid eating too much while others believed the woman would become sick if she did not eat enough. TBAs consistently advocated that pregnant women should not consume fresh milk or eggs because the child may grow too large. Many TBAs said they advised women to consult SBAs for antenatal care, and some actually accompanied them to the dispensary.

During the second trimester, TBAs massage the pregnant woman’s abdomen with oil. Most TBAs recommend food restriction during the second trimester and some TBAs take this to the extreme of instructing women to vomit after eating, with the intention of preventing the baby from becoming too large. Related to this, TBAs also recommend that women recommence domestic work and avoid resting.

*A child can become so big in the womb, the mother will have difficulties during her delivery. That’s why we make her vomit and we don’t give fresh milk or eggs, because the child will become big.* [FGD with TBAs, Laikipia]

In the third trimester, the size of the baby remains a key concern for TBAs so they continue to recommend women take on a high workload, induce vomiting by drinking fermented milk and fat, and in some sites encourage running. If the baby is thought to be large, the TBA may refer the woman for a facility-based delivery. A common practice of TBAs during late stage pregnancy is abdominal massage in the belief that it will correctly position the baby for the birth.

#### Delivery care by TBAs

The role of a TBA during delivery was primarily described by TBAs as “*receiving the baby*” and comforting the mother by “*holding her until she delivers*”. Other women from the community may be present, but the TBA instructs them on what to do. TBAs traditionally feed labouring women oil or animal fat, then herbs, followed by porridge. Women are offered clean water or milk to drink for sustained energy. Abdominal palpation during delivery is practiced by most TBAs to hasten the birth process. Most TBAs allow any birthing position, and women typically express a preference for the squatting position.

#### Post-partum care by TBAs

During the post-partum period, the TBA focuses on thermal care of the mother and newborn, and oversees a rather strict dietary regime for the mother. It is common practice for TBAs to apply a layer of fat to the skin of the mother and the newborn, and wrap them with a cloth to protect from the cold. It is especially important to maintain a warm home as cold is believed to be dangerous for both mother and baby. Immediately after delivery, the TBA ensures that the “*house of the baby*” (placenta) is removed. Following delivery of the placenta, the TBA boils water and bathes the mother, changes her clothing, cooks tea and/or porridge that is fed to the woman, and holds the baby so the mother can rest. TBAs often take over the new mother’s domestic chores such as collecting firewood, managing domestic animals and washing clothes for up to 2–3 weeks after delivery.

The mother’s dietary regime is a cornerstone of the TBA’s traditional knowledge and practice. TBAs usually cook for the woman for the first week or so post-delivery. During the first four days post-partum, women are given meat and soup, tea with milk and sugar, fat and porridge, and every morning and evening are given fresh animal blood to drink. Water is generally withheld and ‘hard foods’ such as rice or beans are not allowed. After one week, the woman is allowed to consume other foods. One TBA described that:

*On the fifth day we give her a traditional herb that cleans the stomach then the following day a ram is slaughtered and (she) is given fat, we give fat to give her diarrhoea and clean out the stomach – it helps her to stop bleeding and after that she is given any hard* [dense/solid] *food*. [FGD with TBAs, Laikipia]

The baby is rubbed with oil, and bathing is delayed for one month or until the umbilical cord stump falls off. In Samburu, some communities reported smearing the cord stump with a mixture of herbs and ashes to stop it from bleeding. Several TBAs said they instruct women to breastfeed immediately after birth, and help them to establish breastfeeding over the ensuing days. Occasional supplementary feeding of the newborn was mentioned; this involved giving water with herbs, and one TBA reported administering boiled water with salt and sugar to a baby for four days when the mother was perceived to have insufficient milk.

#### TBA care in the event of obstetric complications

It is common for TBAs to palpate the woman’s abdomen to assess the term of the pregnancy, predict the sex, and identify potential birth complications such as multiple births, large baby size, or poor positioning of the baby. It is custom in some group ranches for TBAs to tie a “*traditional thread*” loosely around the woman’s waist for spiritual protection if the pregnancy is judged to be high risk*.* In terms of antepartum complications, bleeding is the main concern noted by TBAs, for which they prescribe herbs and animal fat.

For most complications TBAs administer locally collected herbs prepared as a soup or with milk, often combined with fat or animal blood. If this fails, then the mother, reported by TBAs in all group ranches, is referred to a health facility. TBAs identified that some women are “*weak*” or “*do not have enough blood*” (possibly referring to anaemia), and therefore considered to be at high risk of obstetric complications because they may not have the strength to push during labour. Such women are given cow’s blood and often referred for facility-based deliveries.

Retained placenta is a major concern for TBAs. When this occurs they generally induce vomiting by giving the woman large volumes of water to drink.

*We make the woman vomit as this will help the placenta to come out easily and fast. These days, to prevent the placenta from retaining… before cutting the umbilical cord, we make the woman vomit to push the placenta out.* [FGD with TBAs, Laikipia]

For prolonged labour, women are given herbs believed to accelerate labour, and food for additional energy. For breech deliveries, generally referred to as the “*baby sitting in a wrong position*”, TBAs most often refer to a health facility, although in one site TBAs advise women to walk until the baby turns, and the TBAs manage the delivery themselves.

*Children are also delivered by the legs coming out first. There is a way we handle this.* S*ometimes one leg comes out, you push it back, one hand comes again, you push it back, this way we try because there is no hospital near* [FGD with TBAs, Samburu]

TBAs may refer women to an SBA before labour begins for a range of reasons including when the woman is anaemic, very young, giving birth for the first time, expecting twins, experiencing bleeding or has a history of complications or pre-term delivery; or the baby is judged to be too large. More commonly though, referral is only after the TBA attempts to treat the complication herself but is “*defeated*” and realises she cannot manage the situation. All TBAs described referring women for facility-based deliveries for complications occurring during labour. The husband is usually instructed to take the woman to a health facility, at which time the family begins the (often difficult) search for a vehicle. The main indications for referral are prolonged labour (sometimes for days at home), breech presentation, haemorrhage, retained placenta, premature rupture of membranes, cord prolapse, or maternal exhaustion. TBAs often accompany the woman to the health facility, where the majority introduce themselves as a relative rather than identify as a TBA, fearing judgement for not adhering to the Government policy proscribing their practice.

#### Challenges for TBA practice

Managing complications, especially retained placenta, was a major challenge frequently identified by TBAs who commonly acknowledged that SBAs are best at dealing with such problems. Also challenging for TBAs is the difficulty of obtaining transport for women requiring referral to a health facility. Many TBAs expressed concerns about becoming infected with HIV, and consequently conducted deliveries wearing polythene bags on their hands, in lieu of gloves, to protect themselves from becoming “*sick*” (referring to HIV). Several TBAs also mentioned the danger they face when called to attend a delivery that requires them to travel through areas inhabited by wild animals.

*We have to leave our work to help women to deliver. Diseases are many, and if you touch the blood you can be infected. Sometimes we are called at night and there is a lot of wildlife.* [FGD with TBAs, Samburu]

*When we want to take someone to the hospital it is difficult because we don’t have a vehicle. We use polythene bags as gloves, and sometimes you are called far from home at night and you might meet wild animals on your way.* [FGD with TBAs, Laikipia]

### Practices of SBAs in dispensaries

#### Antenatal care by SBAs

SBAs provide a range of services for pregnant women including: assessment of maternal weight, blood pressure, fundal height, foetal heart rate, and foetal positioning. Mothers are issued an antenatal record card, given iron-folic acid supplements and tetanus toxoid injections, and a birth plan is developed. Some health facilities also provide a full antenatal work-up, which includes ultrasound, urinalysis, haemoglobin, blood grouping and HIV testing. Laboratory services are not available at dispensary level, although the nurse may send blood samples to higher level facilities for analysis if the family is able and willing to pay the related costs. SBAs advise women to undertake light work only, increase rest, sleep with an insecticide treated bed net, and consume a balanced healthy diet. From the perspective of community members, antenatal care with SBAs involves administration of injections and medicines, identification of possible complications, and estimation of the delivery date. Several SBAs described involving TBAs in antenatal care – for example in Samburu:

*The moment I meet a mother who is pregnant, I go and tell the TBA “so and so is pregnant, so you monitor her, check if she is coming to the clinic… bring her to the hospital.”* [Interview with SBA, Samburu]

#### Delivery care by SBAs

When a woman presents at a health facility in labour, SBAs assess the status of the mother and baby, determine the stage of labour, and document the woman’s birthing history. SBAs also see it as part of their role to encourage women through the various stages of labour. They try to convince women to deliver in the lithotomy position, a position the women generally dislike. One SBA reported that partographs are used in sub-district hospitals, but are not available at dispensary-level health facilities.

During FGDs with community members, it became clear that in several group ranches, women and their husbands had limited understanding of the practices and services offered by SBAs. It was perceived that SBAs focused mainly on the technical aspects of delivery, and less on supportive care for women. Women who had delivered with SBAs described that they “*make you lie backwards, then tell you to push out, and she makes a lot of noise for you until you give birth, while she is just standing there looking at you”* [FGD with community women, Laikipia].

SBAs working at dispensary level attempt to manage obstetric complications, but readily call a vehicle and escort the mother to a higher level facility (district hospital) if indicated. Some SBAs also informally collaborate with TBAs at the time of delivery.

*We must have the TBAs here* [in the dispensary]. *They accompany the women so they can feel at least I have someone of my own with me – I have a TBA around. So we encourage them – if you are in labour, come to the hospital and let the TBA accompany you.* [Interview with SBA, Laikipia]

#### Post-partum care by SBAs

After birth, SBAs routinely examine the newborn, wash the mother, and show her the baby. A woman typically remains in the facility for 24 h following delivery, with both mother and baby receiving a check-up prior to discharge. SBAs monitor the woman for post-partum bleeding, provide breast feeding advice, counsel her regarding family planning, and explain the necessary care of episiotomy wounds or vaginal tears. Women are advised to eat plenty of food, and if a mother is underweight she may be given flour and beans to supplement her diet. Family visits are restricted to visiting hours only, unless there is a complication, in which case the family is allowed greater access. Some SBAs arrange for CHWs to provide home visits for post-partum women.

#### Challenges for dispensary-based SBA practice

SBAs identified a number of challenges in relation to their work. Many found it particularly difficult to convince women to deliver in the health facility. Even women who are willing to attend for antenatal care, prefer to labour and deliver at home, only using the health facility if they experience complications. Many SBAs described requests to deliver women in their own homes and felt duty-bound to respond. This involves finding a means of transport or walking long distances, sometimes through areas inhabited by wild animals, and the health facility is left unattended while the SBA attends the home birth.

*They call us to go and deliver them at the manyattas, which are very far away, and the mode of transport is very poor, and we come across wild animals on our way.* [Interview with SBA, Laikipia]

Several SBAs expressed concern regarding the infection risks for mother and baby in the relatively unclean home environment, and the frustration of being called to attend a woman at home who is already in advanced labour and experiencing complications.

Another major challenge for SBAs is obtaining an ambulance for women who urgently need to be transferred to higher level facilities. When phoning for an ambulance, sometimes no-one answers, the SBA is told that there is no driver or no fuel available, or advance payment for fuel is demanded from the woman’s family before the ambulance will be dispatched. Several SBAs reported that their dispensaries lacked basic essential equipment and medicines, making it very difficult for them to practice in a skilled and safe way. Being a lone practitioner with no colleagues to double-check assessments or share decision-making was concerning for several SBAs, especially in the event of obstetric emergencies. Others mentioned the challenge of one person having to fulfil a number of different roles, and deliver on both government and community expectations.

Dispensaries are left unattended when SBAs travel for work-related or personal reasons. When labouring women arrive at the facility seeking skilled birth attendance and the SBA is absent, they and their family members become (understandably) very annoyed, and do not always understand that the nurse’s absenteeism may be legitimate.

*I am the only one there. If I go for a seminar the facility is left unattended. So that is one of the challenges – the workforce is very low… We have a lot of work, we are the clinical nurse, we are everything… We are given accounts, we are the manager of the whole facility, and we have to work with the community… The community does not understand… If they find that the nurse is not in the facility, they just say she is not at work. Even though I am at a seminar or have taken some official things to town, they will just say ‘the nurse is not there.’* [Interview with SBA, Samburu]

### Perceptions of TBAs

#### Strengths of TBAs

According to TBAs, their primary strength is their availability to the community. They are always available to assist, and commented that they had delivered many of the people in their communities over a long period of time, including those who are now SBAs. They see themselves as particularly useful for young mothers, and when nurses are not available. They value their own traditional knowledge, particularly their expertise regarding herbs and foods that should be consumed (or not) around the time of pregnancy and childbirth. They see this as knowledge that doctors do not possess. Many community members and TBAs described how it is necessary to have courage to be a TBA.

From the perspective of SBAs, the strength of TBAs is the emotional and practical support they provide to mothers. SBAs report that some TBAs advise women to attend the health facility for antenatal care, accompany them for facility-based deliveries, and promote immunisations. TBAs are considered particularly important in remote areas where health facilities are inaccessible, particularly if they have delivery kits and are able to assist women unable to otherwise reach facilities. Some SBAs perceived the traditional knowledge of TBAs as an admirable attribute. Several SBAs mentioned that TBAs are widely trusted, and have a very comfortable communication style with the women. Overall, SBAs perceive TBAs as an asset to the health system because of their influence in the community.

Community members were very affirming of the TBA role, and clearly trusted them and held them in high regard.

*Women want to deliver at home because they see that a TBA will deliver them well. She is the one who will light a good fire, she will clean her well, so you find that the majority like to deliver at home.* [Interview with female CDC member, Samburu]

The fact that TBAs are available, accessible and affordable was particularly valued by community members. Additionally, there are no transport challenges or transport costs incurred when women are cared for by TBAs. Community members mentioned that TBAs accompany women to health facilities where possible, and are essential in remote communities where health facilities are inaccessible. The traditional knowledge held by TBAs was considered a valuable asset. Women and their husbands trusted TBAs’ abilities to determine the position and sex of the baby, massage the women’s abdomen to position the baby for birth, and administer herbal remedies. Community leaders said that TBAs were particularly helpful for first-time mothers who were less likely to fear a TBA, and would not feel shy about sharing the “*secrets of the body*”.

#### Concerns regarding TBAs

The main concern regarding TBAs was their inability to manage the complications of childbirth. This was echoed by all categories of participants, although the views expressed varied somewhat. Community members were of the opinion that TBAs do know about likely complications, but are unable to manage them because they lack the training and equipment. In contrast, SBAs maintained that TBAs are not competent to recognise complications such as prolonged labour, anaemic mothers, and premature separation of the placenta, and therefore delay referring women to health facilities. SBAs and CHWs both pointed out that TBAs cannot manage post-partum complications such as retained placenta and haemorrhage, nor can they administer injections or undertake any surgical procedures.

Unhygienic practices of TBAs were frequently mentioned as a major concern, voiced by TBAs themselves, women, SBAs and community leaders. This was linked to the TBAs’ lack of training and resources (delivery kits), making it difficult for them to provide sanitary care. The absence of hand washing and gloves, dirty utensils used for cord cutting, and the generally unclean delivery environment were noted as potential causes of disease affecting both mothers and newborns.

*Some are dirty and they don’t even wash their hands. Some don’t have the knowledge, and they can use anything to cut the umbilical cord, they force you to push out the baby before time.* [FGD with women delivered with no TBA or SBA, Samburu]

SBAs identified several TBA practices as dangerous including: encouraging women to push before the cervix was fully dilated; allowing delivery in the squatting position (which SBAs linked to complications); neglecting the delivery of the placenta and postpartum haemorrhage; administration of herbs; inducing vomiting; and instituting restrictive ante- and post-partum dietary regimes. SBAs were of the firm opinion that TBAs should not attempt to deal with complications as this only delays subsequent referral for assistance, and some SBAs believed that delays in referral are in part because TBAs want to avoid loss of payment for the delivery.

### Perceptions of SBAs

#### Strengths of SBAs

From their own perspective, the primary strength of SBAs is their technical knowledge, skills and experience. They see themselves as able to detect women and newborns at risk of complications, and having the skills and medicines to manage them. Beyond the skills of individual SBAs, being a part of the health system was considered valuable because it provides a safe environment for conducting deliveries, and means that SBAs are more easily able to refer women to higher level facilities if unable to handle the situation themselves.

From the perspective of TBAs, the strengths of SBAs lie in the resources and skills they have. TBAs respect SBAs’ training, and appreciate it when they are willing to share information and advice. They admire SBAs’ ability to remove retained placenta, manage multiple births and determine if a woman is HIV positive. SBAs are seen to have an advantage because they possess medicines and equipment (such as gloves), are able to administer injections, and can perform caesarean section if indicated (although this is not actually available at dispensary level or even sub-district level).

Community members were of the view that SBAs possess specialised knowledge and are particularly valuable for complicated deliveries such as twins, retained placenta or if the mother is “*sick*” (HIV positive). Community members also identified the injections given by SBAs following delivery as a strength, although there were varied opinions regarding the benefit of these injections. For instance, they were thought to prevent bleeding, reduce labour pain, prevent the woman from feeling cold, “*wash the womb*” and prevent “*stomach problems*”. Other strengths identified were the capacity to perform caesarean section, dispense medications, and refer to higher level facilities.

*When you go there with different problems, they [SBAs] can treat you. It’s good they help if you have difficulty with the first child. You are given injections to prevent bleeding. You don’t bleed like at home.* [FGD with women delivered by TBAs, Samburu]

Dispensary-level services were preferred over sub-district and district health facilities as they are nearer, and dispensary nurses are considered to be more attentive than nurses in hospitals, as they do not have too many patients at one time. Across group ranches, community members welcomed the fact that SBAs, unlike TBAs, allowed women to eat any food they want. The SBAs were said to have brought hope, as one community leader said:

*If the nurse is not near…these people, how will they be? They will just die. We see the goodness of the doctors and nurses being near the community. The community has hope.* [Interview with female CDC member, Samburu]

#### Concerns regarding SBAs

Concerns about SBAs, mentioned by TBAs, community women and even by SBAs themselves, pertained to the negative attitudes and behaviours of some SBAs. Experiences of poor quality care and dissatisfaction with care were reported by several women – some accounts were of personal experiences and others were descriptions based on anecdote. Women reported verbal and physical abuse by SBAs including insults, slapping, pinching and being clipped with scissors. These experiences were mainly associated with higher level health facilities (not dispensaries), where they described being left unattended, abandoned, and neglected.

*Only those who are courageous go to the hospital. I can’t go because I am afraid. I deliver at home so I can get someone to hold me. In hospital you are beaten, at home you get someone who comforts you until you deliver.* [FGD with TBAs, Laikipia]

*Women are not comforted, you are left alone. Doctors keep a distance and keep watching you. You are also tied. During labour pains you meet a doctor who slaps you seriously. At home you say let me stay where nobody beats me up. Some don’t want to be stitched and you are told to remove your clothes.* [FGD with TBAs, Samburu]

Some SBAs were considered to be arrogant and unmotivated, and appeared to treat women’s presence in their health facility as an inconvenience. Some women fear health facilities, particularly large hospitals, citing concerns that: children can be swapped or exchanged; women and babies don’t return from there (because they have died); unfamiliar medicines are administered; birthing beds are too high; and facilities are too cold, which is considered to be dangerous to the health of mothers and babies. Some women do not attend SBAs because they want to avoid certain clinical procedures, especially episiotomy and suturing, vaginal examination, surgery, being forced to deliver in the lithotomy position, and being without clothing.

These negative perceptions of SBAs were at odds with SBA’s views of themselves. Without exception, the SBAs readily recognised that good quality care is characterised not only by technical competence but also by kind, respectful and patient communications with people they care for.

*You have to be patient and tolerant. Then you must be friendly to them… Talk to them, share some stories, make them laugh. Share some experiences you have had.* [Interview with SBA, Laikipia]

*You just have to be gentle. You just have to be tender and caring. Not just standing there, you should be attending to that woman.* [Interview with SBA, Laikipia]

## Discussion

The findings from this qualitative study describe the practices of traditional and skilled birth attendants providing care for pastoralist communities in Laikipia and Samburu counties, Kenya, as well as the perceptions of the value and limitations of SBAs and TBAs from a range of perspectives including those of the practitioners themselves, the communities they serve, and key informants from the formal health system and from the Community Development Committees.

TBAs are described as familiar, trusted and respected repositories of traditional knowledge, who are actively engaged with pastoralist women during pregnancy, birth, and especially post-partum. Several TBAs have built links with SBAs in their areas, and are encouraging women to attend the dispensary. However, despite these positive attributes, it is probable that some routine TBA practices are potentially harmful to women and their babies, especially dietary advice involving food restrictions, induction of vomiting, abdominal palpation/massage, administration of herbs, and inadequate personal and environmental hygiene. It is widely acknowledged by participants (including TBAs) that TBAs do not possess the knowledge, skills, or equipment required to recognise and manage obstetric and neonatal complications. When complications occur at home, arranging referral and transport to the health facility is a challenging process for all involved, likely to result in substantial delays.

SBAs are recognised by all categories of participants to have the technical skills and resources required to provide safe and hygienic deliveries. However, SBAs regularly encounter a number of challenges that potentially compromise the quality of care they provide. Clearly, many pastoralist women remain reluctant to deliver in the health facilities, even if they have engaged with antenatal care. The health facilities are thought to be cold, unfriendly and even frightening, which possibly deters women from attending. Similarly, the reputation of SBAs as uncaring, and even abusive, is likely to negatively influence the uptake of health facility services. There is growing international awareness of this problem and the urgent need to address it in order to increase the proportion of births attended by SBAs, as evidenced by the 2014 WHO statement on ‘The prevention and elimination of disrespect and abuse during facility-based childbirth’ [16].

The SBAs are often practicing alone, which makes it difficult for them to staff the dispensaries at all times, and occasional absenteeism can be judged harshly by the community. Managing complicated deliveries as a solo practitioner is stressful, and arranging transfers to higher level facilities is not always a smooth process. Additionally, dispensaries are not always adequately equipped with essential items such as birthing kits and medicines. Despite these issues, there is ample evidence of mutual respect between SBAs and TBAs, and it is clear that some are already collaborating for collective benefit in the provision of care for women and babies in these remote settings. Creating greater collaboration between SBAs, TBAs and communities may maximise the strengths, fill the gaps and address the concerns regarding both SBA and TBA practices.

The development of a model for SBA/TBA collaborative care is likely to increase the uptake of SBA services [4, 10, 17]. For example, in a rural county of Kenya, TBAs were trained to provide birth planning advice and given a financial incentive for referrals to SBAs, which resulted in a 113 % increase in facility-based deliveries [18]. Evidence suggests that more collaborative models of care that collectively engage SBAs, TBAs and communities can improve the quality of maternal and newborn care and the utilisation of health services, as well as avert obstetric complications and perinatal, newborn and maternal mortality [11, 18–21].

However, it is critical to recognise that other persistent barriers such as distance and accessibility, staff availability, cost of services and transport, informed decision making, social and cultural preferences, must also be addressed [11, 19].

In the context of the pastoralist communities in Laikipia and Samburu, TBAs could, and in some places already do, escort women to dispensaries for antenatal care, refer them to the SBA at the onset of labour, provide them with support and comfort during delivery, and assist with domestic chores pre- and post-partum. Women may be more inclined to attend health facilities for maternal care if they are accompanied by a trusted, respected member of their own community, and the provision of continuous support to women in labour results in better obstetric outcomes and a more positive birth experience [22]. Additionally, TBAs could assist SBAs, who are predominantly solo practitioners, at the time of delivery. TBAs would require basic training to facilitate this new role, and to eliminate some of their more unsafe practices. If TBAs escort labouring women to health facilities, their presence will ensure that women are not left alone. The approach of engaging female birth companions from local communities, such as TBAs, has been assessed in 13 randomised controlled trials and found to reduce the incidence of adverse birth events and improve satisfaction with facility-based delivery among mothers [23].

This study has certain limitations that should be considered when interpreting the findings. Although data collection was designed to capture a range of perspectives from the sampled communities, as with all qualitative research, the findings are not representative of these communities, and cannot be generalised. Social desirability bias may have influenced some responses i.e., what participants actually believe, would do or have done may differ from what they reported. The SBAs in our study were based at dispensary level health facilities and therefore quite familiar with the communities and TBAs. Their views about TBAs may not be shared by SBAs based in higher level facilities such as district hospitals.

## Conclusion

These findings suggest that a collaborative model of care involving both TBAs and SBAs is a viable proposition. While the Government of Kenya’s goal is 90 % skilled birth attendance, the current reality among the semi-nomadic pastoralist communities in Laikipia and Samburu is that the majority of births are occurring at home with TBAs in attendance. The transition from predominantly traditional birth to skilled birth attendance in these communities is going to be a gradual one, and an interim collaborative model has the potential to maximise the safety of pastoralist women and babies during the transition phase, as well as facilitating the transition itself.

## Abbreviations

CDC: Community Development Committee
CHW: community health worker
FGD: focus group discussion
IDI: in-depth interview
MUACK: Mothers’ Union of the Anglican Church Kenya
SBA: skilled birth attendant
TBA: traditional birth attendant
